# Supplementary material for: An FcRn-targeted mucosal vaccine against SARS-CoV-2 infection and transmission
Source: Nat Commun. 2023 Nov 6;14:7114. doi: 10.1038/s41467-023-42796-0 (PMC10628175; doi:10.1038/s41467-023-42796-0)
Supplement: Supplementary file 1 — Supplementary Information [file 41467_2023_42796_MOESM1_ESM.pdf]

An FcRn-targeted mucosal vaccine against SARS-CoV-2 infection and transmission

Li et al. 2023

Supplementary information

Supplementary Figures

Supplementary Tables

Supplementary Notes

Supplementary Figures

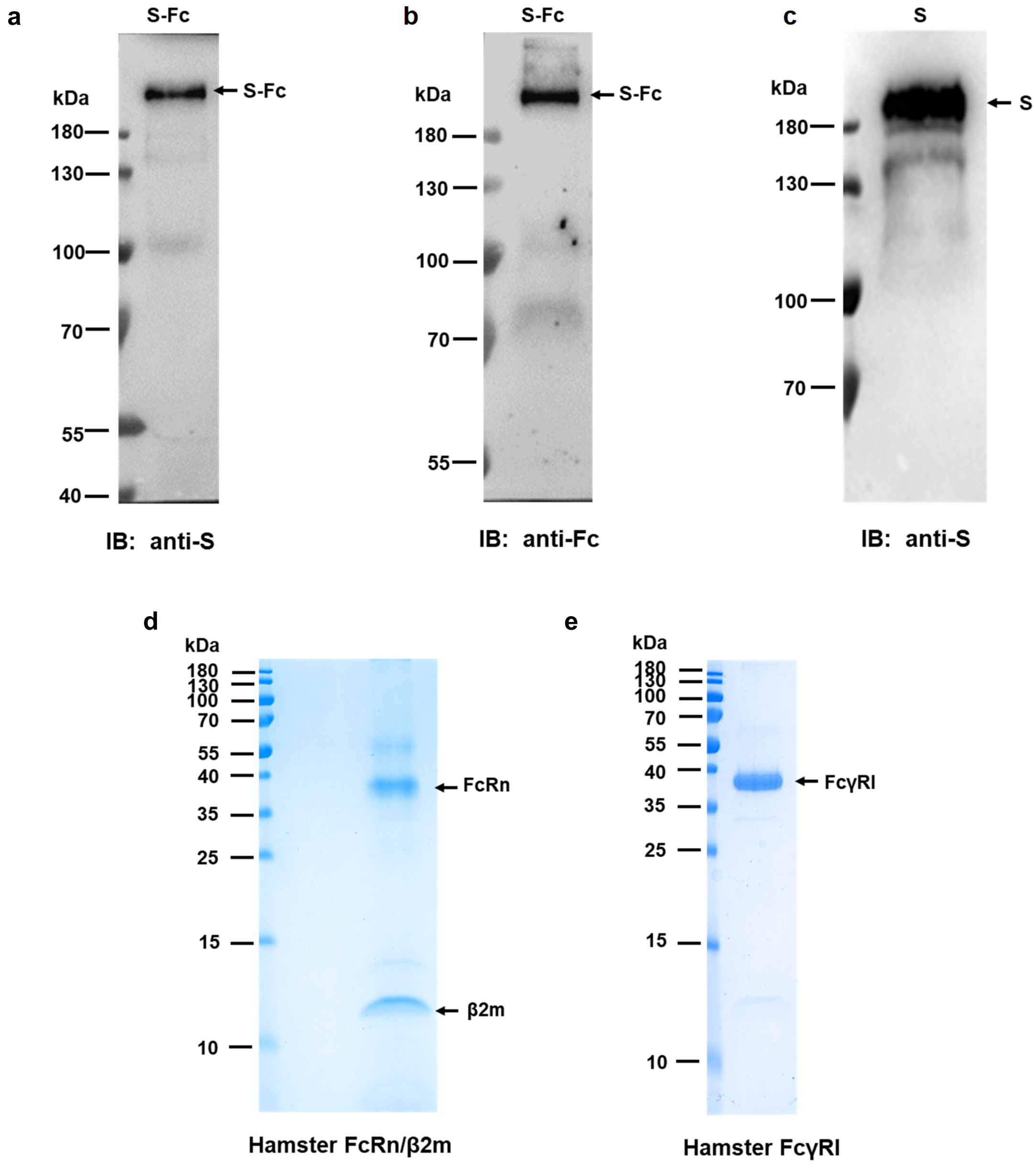

Supplementary Figure 1 Expressions of S-Fc, S, hamster FcRn/β2m, and FcγRI proteins. The S-Fc fusion protein (**a**, **b**) or S (**c**) protein purified from the stable CHO cell line was identified by Western blot. The S-Fc or S proteins were subjected to SDS-PAGE and Western blot analyses and detected by either anti-S (**a**, **c**) or goat anti-human IgG-Fc Abs (**b**). HRP-conjugated secondary Abs and the ECL method visualized the S-Fc or S proteins. The purified hamster FcRn/β2m proteins (**d**) or hamster FcγRI proteins (**e**) from the recombinant plasmid-transfected 293T cells were visualized by Coomassie blue staining.

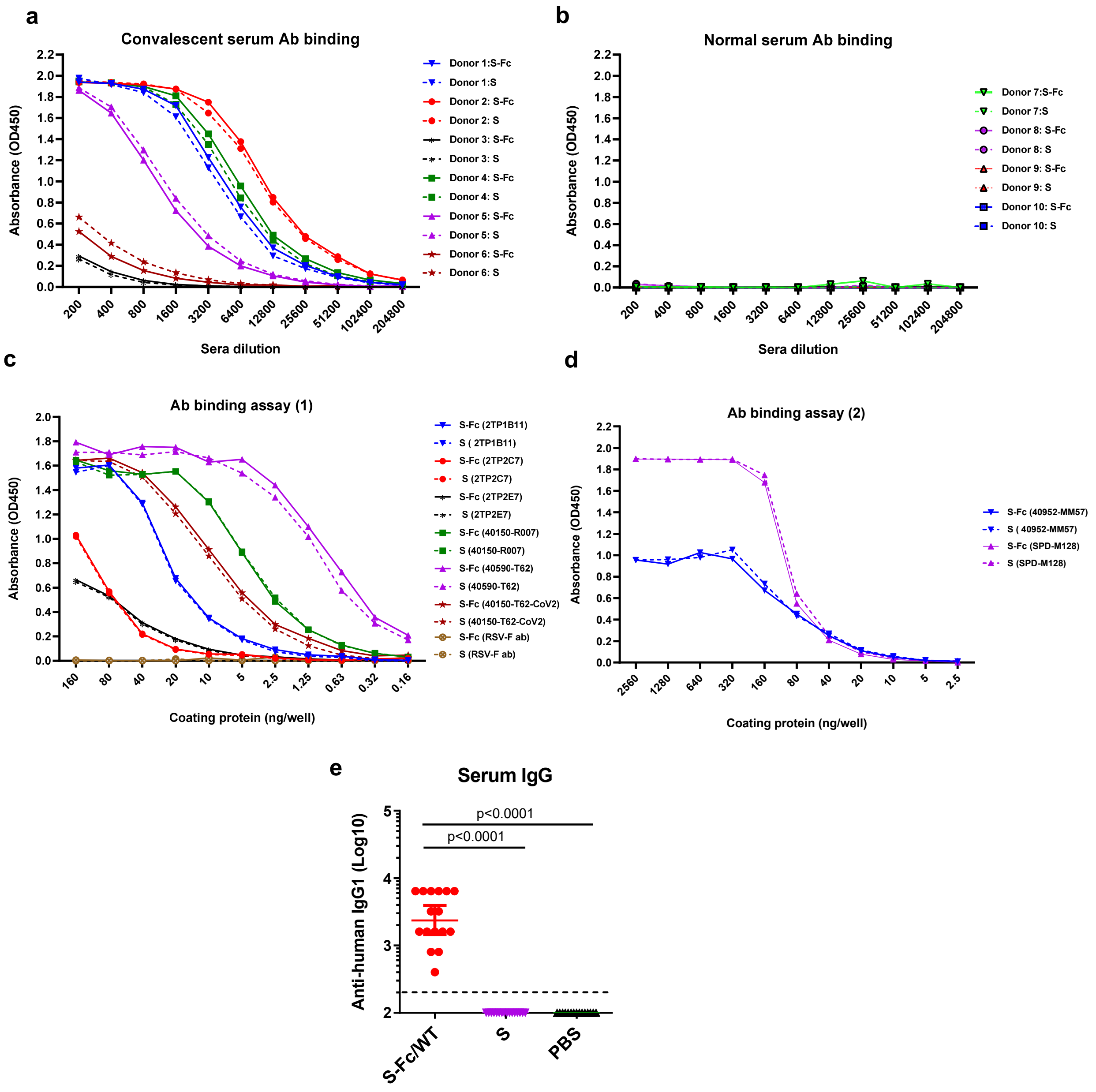

Supplementary Figure 2 a-d Interactions of the purified S or S-Fc with S-specific antibodies. Interactions of the purified S or S-Fc with COVID-19 convalescent human serum (**a**), normal human serum (**b**), and a set of SARS-CoV-2 S-specific mAbs (**c**, **d**). The specific binding was detected by the ELISA method. **e** Anti-human IgG1 Fc-specific IgG Ab titers in mouse sera. Ten µg of S-Fc, S (with the equivalent molar number), or PBS in combination with 10 µg of CpG was i.n. administered into 6-8-week-old wild-type (WT) C57BL/6 mice. Mice were boosted 14 days after the primary immunization. The IgG1 Fc-specific Ab titers were measured by coating the plates with human IgG1 in ELISA. The IgG titers in mouse sera (n=16 for S-Fc/WT and S groups, N=15 for PBS group) were measured. The data represent a geometric mean with 95% CI. Statistical differences were determined by one-way ANOVA (Kruskal-Wallis test followed by Dunn's multiple comparisons test). Dashed lines indicate LOD.

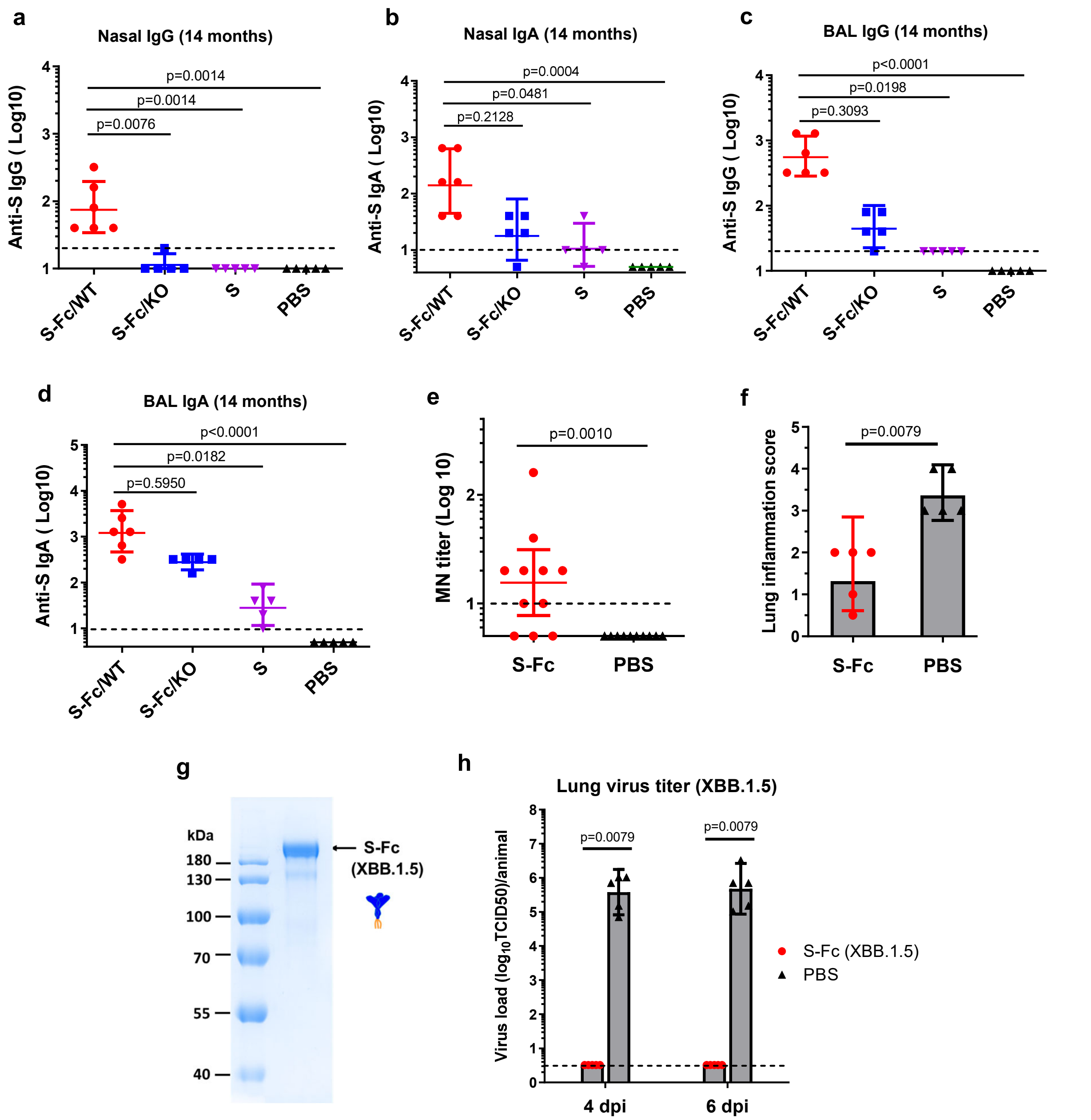

Supplementary Figure 3 **a-d** Anti-S-specific Ab titers in nasal washings (**a**, **b**), and BAL (**c**, **d**) after 14 months of the boost. ELISA measured SARS-CoV-2 S-specific Abs from mouse samples (n=6 for S-Fc/WT group, n=5 for other groups). The data represent a geometric mean with 95% CI. **e** SARS-CoV-2 Delta-specific nAbs in human ACE2 mice after the boost. The micro-neutralization test determined the nAb activity in the sera (n=11 for S-Fc/WT group, n=10 for PBS group). The data represent a geometric mean with 95% CI. **f** After the Delta strain challenge, the inflammatory responses of each lung section were scored *blindly.* **g** The S-Fc fusion protein derived from Omicron XBB.1.5 strain was purified from the 293F cell line. The S-Fc proteins were purified by Protein A affinity column, subjected to SDS-PAGE gel

electrophoresis under reducing conditions and visualized with Coomassie blue staining. The molecular weight in kDa is marked in the left margin. **h** Viral titers in the lungs 4 and 6 days after the challenge of Omicron XBB.1.5 (5 x 10^4 $TCID_{50}$). Supernatants of the lung homogenates were added to VAT cells and incubated for 4 days. The viral titers were shown as $TCID_{50}$ from each lung. Statistical differences were determined by one-way ANOVA (Kruskal-Wallis test followed by Dunn's multiple comparisons tests) (**a-d**) and Mann Whitney test (two-tailed) (**e**, **f**, **h**). Dashed lines indicate LOD.

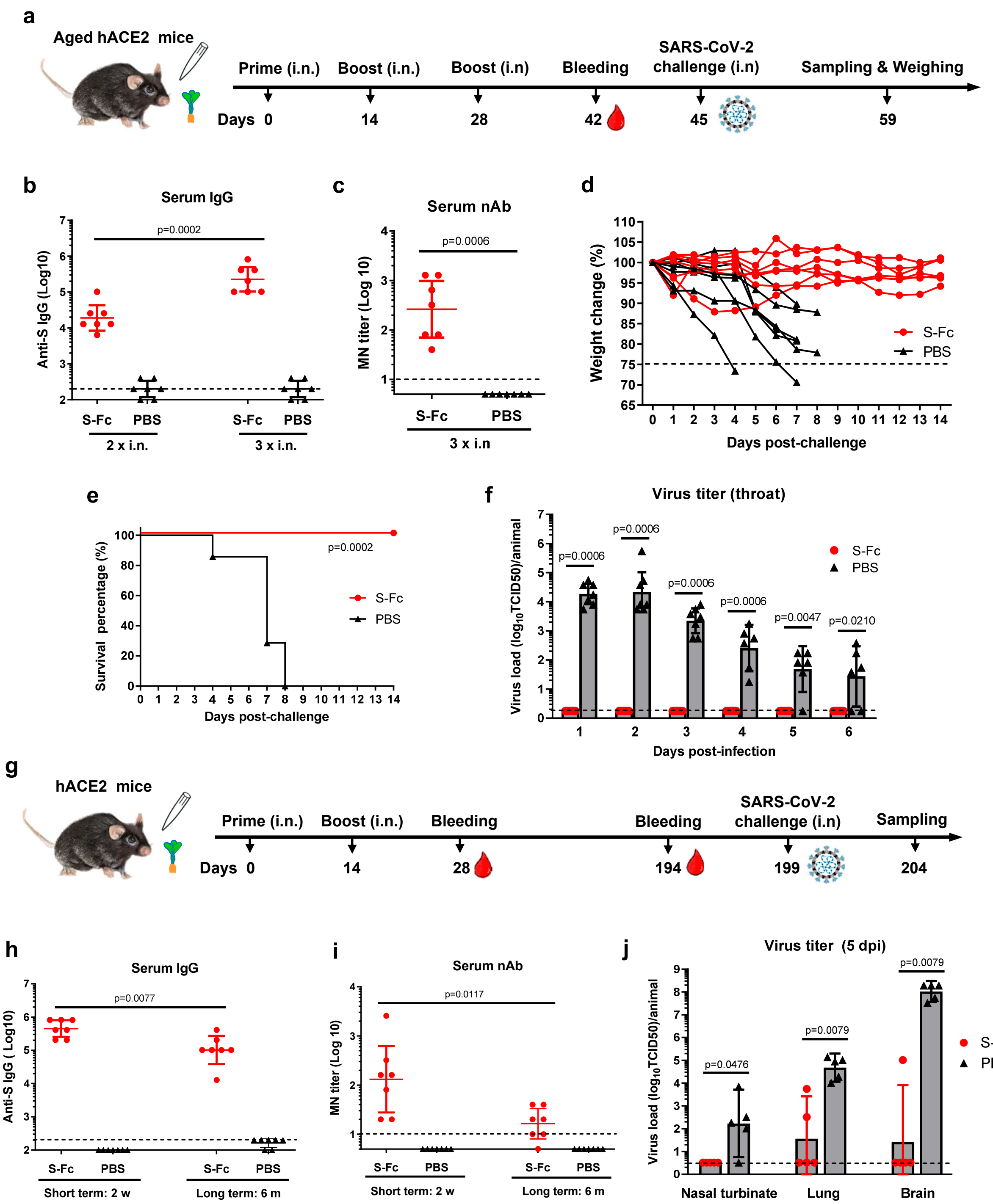

Supplementary Figure 4 Intranasal immunizations with the S-Fc induce protection in old mice and a protective memory immune response. a The 12-18 months-old hACE2 mice were i.n. immunized by 10 μg S-Fc or PBS with 10 μg CpG and boosted twice at a 2-week interval. **b** Anti-S-specific IgG Ab titers in the old mouse sera (n=7 per group) were measured by ELISA

14 days after the first- and second-time boosts. 2 x: one-time boost; 3 x: two-times boost. **c** The nAb titers against ancestral SARS-CoV-2 were measured 2 weeks after the second boost in the old mouse sera (n=7). **d** Body-weight changes following the challenge. Seventeen days after the second boost, the old mice (n=7) were i.n. challenged with ancestral SARS-CoV-2 virus (5 X 10^3 $TCID_{50}$) and weighed daily for 14 days. **e** The percentage of the old mice protected on the indicated days was shown by the Kaplan-Meier survival curve. **f** Throat samples were collected daily for 6 days in each aged mouse after the challenge. The presence of live virus ($TCID_{50}$) from each animal swab was measured in VAT cells after 4 days. **g** The 8-week-old hACE2 mice were i.n. immunized by 10 μg S-Fc or PBS with 10 μg of CpG and boosted once in a 2-week interval. Six months after the boost, mice were i.n. challenged with the ancestral SARS-CoV-2 (2.5 X 10^4 $TCID_{50}$). **h** Anti-S specific IgG Ab titers in sera (n=7) two weeks (2 w) or six months (6 m) following the boost were measured by ELISA. **i** The nAb titers in sera from the immunized mice (n=7) were determined using the $TCID_{50}$ test 2 weeks or 6 months after the boost. **j** Viral titers (n=5) were measured in the nasal turbinate, lung, and brain at 5 dpi. The data represent a geometric mean with 95% CI for **b, c, f, h, I, and j**. The statistical analyses were performed by the Unpaired T-test (two-tailed) (**b, h, i**), Mann-Whitney test (two-tailed) (**c**, **f**, **j**), and Log-rank (Mantel-Cox) test (**e**). Dashed lines indicate LOD.

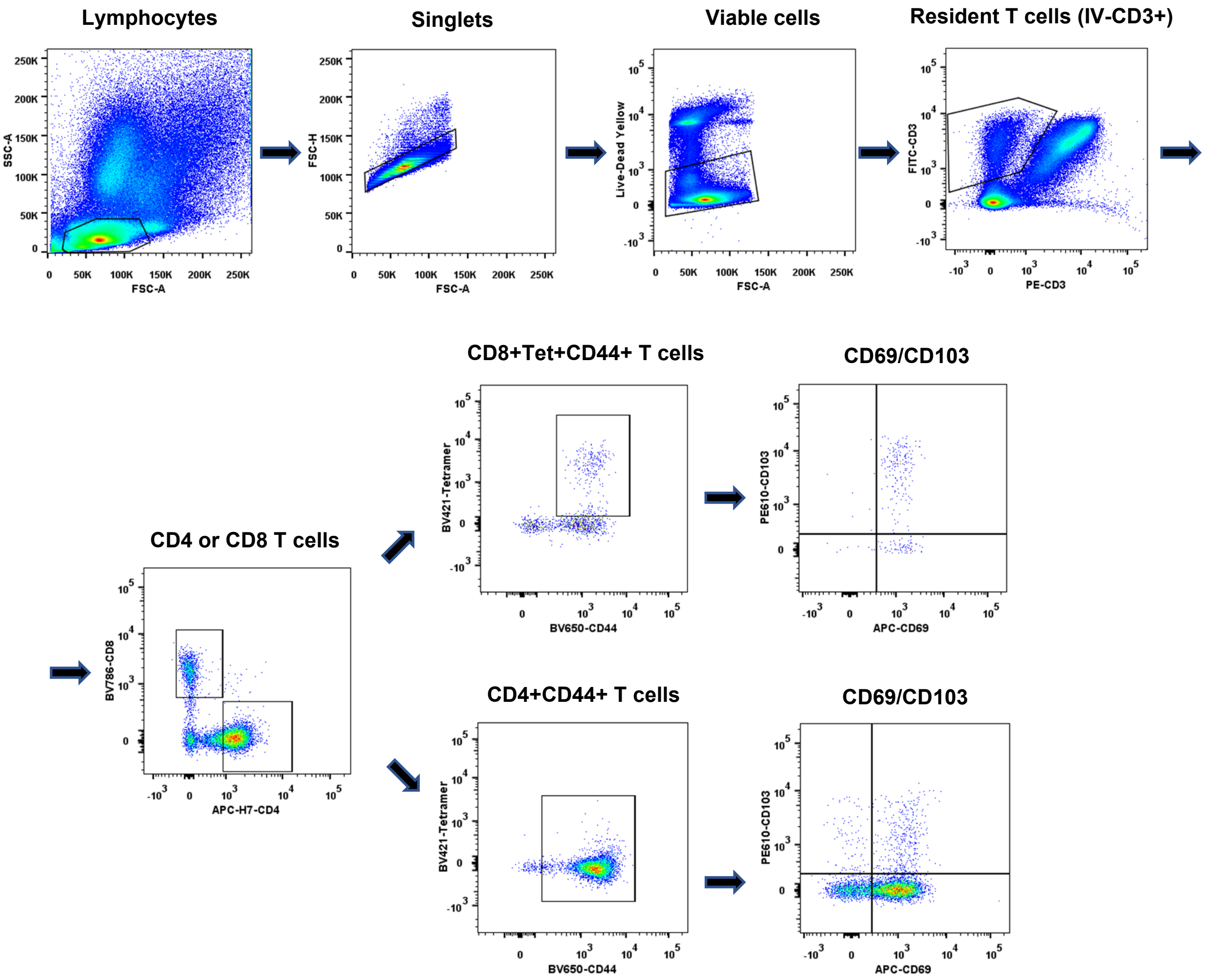

Supplementary Figure 5 Gating strategy for identifying TRM T cells in the lungs. The lymphocyte population was first gated from the lung tissue's mixed cells in the plot of FSC vs. SSC. Then an FSC-H vs. FSC-A plot was used to select singlets, followed by a viability dye staining to exclude the dead cells. An intravenous (IV) staining strategy using PE-CD3 was applied to distinguish resident cells from others to rule out the positively stained circulating T cells. The negative population (IV-) showing the positive reaction for the in-vitro FITC-CD3 staining was characterized as a T subset in the lung. This subset was further separated into $CD4^{+}$ and $CD8^{+}$T cells and subjected to the CD44-Ab staining and S-specific tetramer staining. Finally, after the staining with antibodies against CD69 or CD103, the representative phenotypes of TRM were defined.

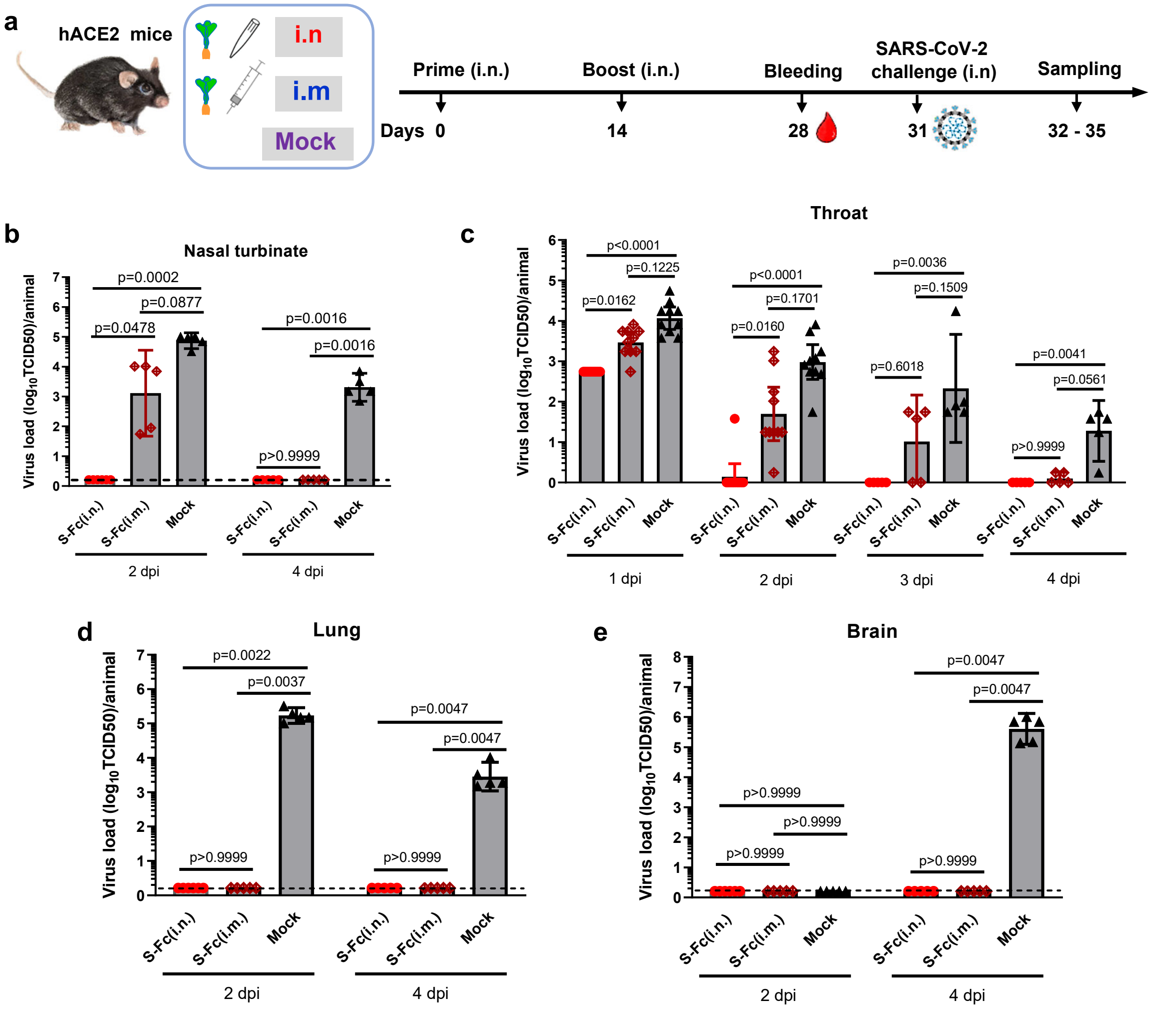

Supplementary Figure 6 FcRn-mediated intranasal vaccination reduces viral replications in the upper respiratory tract. a. Ten µg of S-Fc with 10 µg of CpG was i.n. or i.m. administered into 6-8 week-old hACE2 mice (n=11 for i.n group and n=10 for i.m group). Mice were boosted 14 days after primary immunization. A group of mice (n=10) were mock immunized as a negative control. Throat swabbing was performed daily from 1- 4 dpi. Half mice in each group were euthanized at 2 and 4 dpi, respectively, for harvesting tissues and titrating the virus. **b-e** Samples were collected from throat swabs, nasal turbinates, and lung and brain tissues at multiple time points after the challenge, as displayed at the bottom. The live virus titers were determined by calculating $TCID_{50}$ values. The data in all figs represent a geometric mean with 95% CI. One-way ANOVA (Kruskal-Wallis test followed by Dunn's multiple comparisons tests) were used for the statistical assay (**b,c,d,e**). Dashed lines indicate LOD.

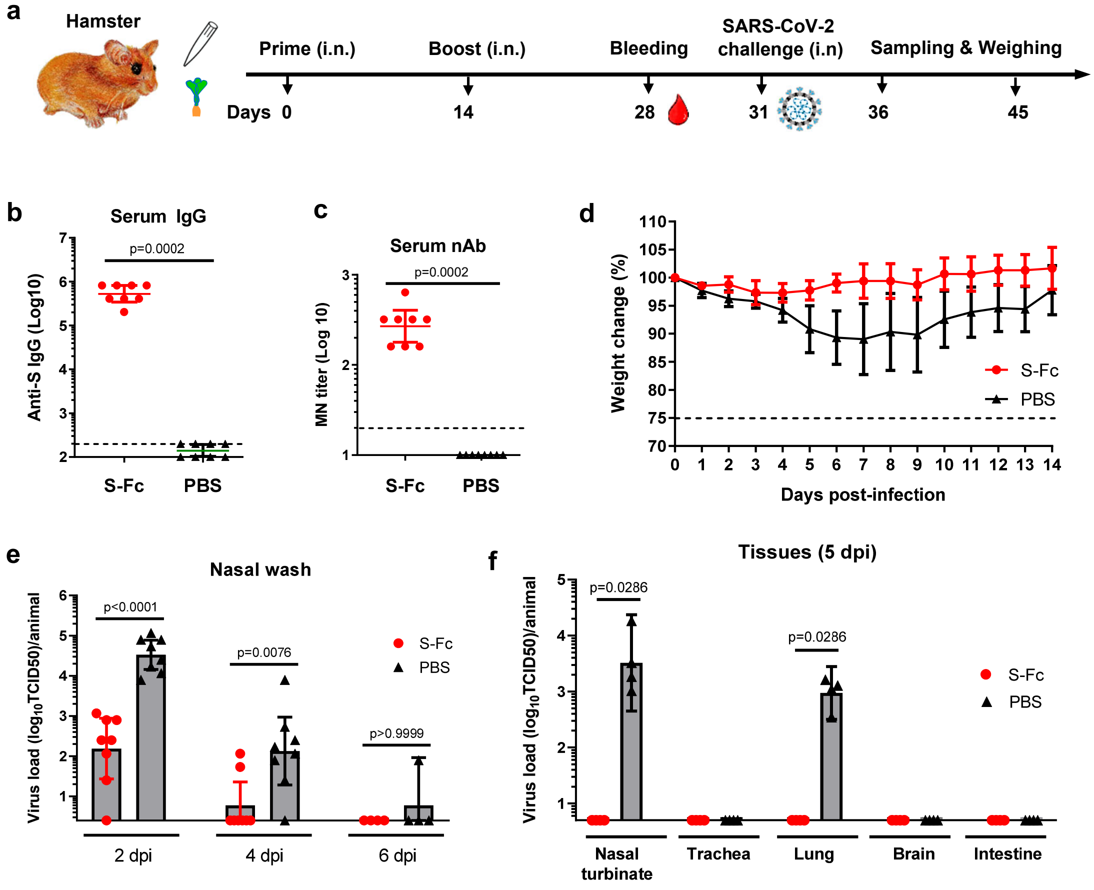

Supplementary Figure 7 Intranasal immunizations with the S-Fc protein protect *Golden Syrian* hamsters from SARS-CoV-2 infection. a 30 μg of the S-Fc (n=8) or PBS (n=8) in combination with 30 μg of CpG was i.n. administered into female hamsters twice in a 2-week interval. Animals were challenged with ancestral SARS-CoV-2 (1 x 10^5 $TCID_{50}$), and nasal washings were collected at 2, 4, and 6 dpi. Four hamsters from each group were euthanized and sampled at 5 dpi for titrating the virus. The 4 hamsters in each group were used for weight loss and survival assay. **b** Anti-SARS-CoV-2 S-specific IgG Ab titers in the hamster sera. The S-specific Ab titers were measured by coating with S protein in ELISA 14 days after the boost. The IgG titers from eight hamster sera per group were calculated. **c** The nAb in the immunized hamster sera. Two weeks after the boost, sera sampled from eight hamsters per group were heat-inactivated and serially diluted two-fold in PBS. The micro-neutralization test determined the neutralizing Ab activity in the sera. **d** Changes in body weight after virus challenge. Seventeen days after the boost, hamsters were i.n. challenged with 1 × 10^5 $TCID_{50}$ of ancestral SARS-CoV-2 and weighed daily for 14 days. Hamsters were euthanized at the end of the experiment or when a humane endpoint was met. The data represent mean ± SD. **e** Shedding of SARS-CoV-2 virus in nasal wash samples of the immunized and controlled hamsters. The virus levels were determined by a $TCID_{50}$ assay. **f** The viral titers in the nasal turbinate, trachea, lung, brain, and

intestine at 5 dpi. Four animals from each group were euthanized for virus titration 5 days after the challenge. Supernatants of the nasal turbinate, trachea, lung, brain, and intestine homogenates were added onto Vero-E6 cells and incubated for four days. The data in **b,c,e,f** represent a geometric mean with 95% CI. The viral titers were shown as $TCID_{50}$ from each animal swab. The statistical analyses were determined by Mann-Whitney test (two-tailed) (**b, c**, **f, e** at 6 dpi) and unpaired T-test (two-tailed) (**e** at 2 dpi and 4 dpi). Dashed lines represent LOD or humane endpoint (D).

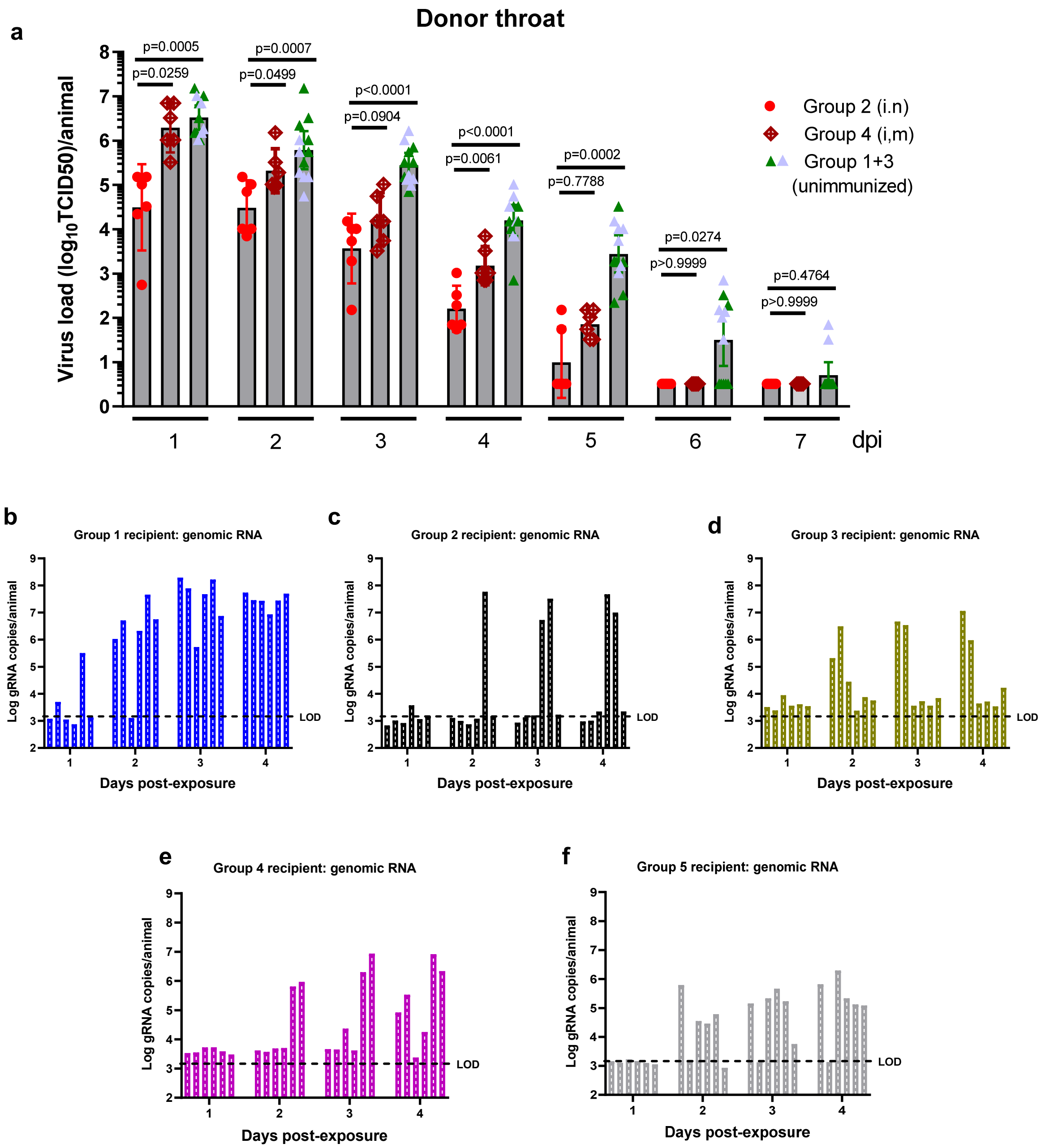

Supplementary Figure 8 Comparison of virus load and RNA levels in the throat swab. **a** Comparison of virus load in the throat swab of the donor hamsters from different groups after the SARS-CoV-2 challenge (n=6 for group 2 and 4, n=12 for group 1+3). The presence of the infectious live virus 1-7 days after infection is determined by $TCID_{50}$ assay in VAT cells and shown as geometric mean with 95% CI. The ordinary one-way ANOVA followed by Dunnett's multiple comparison test was used for statistical analysis of the data collected at 2, 3, 4 dpi, while Kruskal-Wallis test followed by Dunn's multiple comparisons tests was used for data collected 1, 5, 6, and 7 dpi. **b-e** SARS-CoV-2 RNA levels in throat swabs. Total RNAs were

isolated and subjected to the one-step qRT-PCR analysis. Viral loads were quantified as SARS-CoV-2 N gene RNA in throat swab fluid on days 1-4 after exposure. Viral RNA was expressed as N gene RNA copy numbers from each swab of animal, based on an RNA standard included in the assay. Each bar represents one animal. Dashed lines indicate LOD.

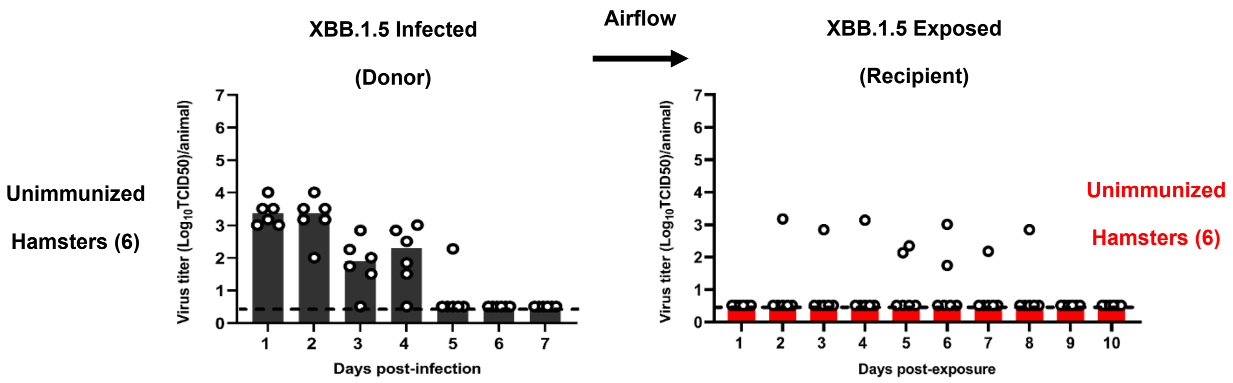

Supplementary Figure 9 Transmission of Omicron XBB.1.5 subvariant in hamsters. The donors (n=6) or recipient (n=6) hamsters were unimmunized. The donor hamsters were i.n. infected with 1×10^5 $TCID_{50}$ Omicron XBB.1.5 subvariant. Fourteen hours later, donor hamsters in the wire cages were separately cohoused with 6 recipient hamsters within the same isolator. Throat swabbing was performed for 7 days for donor hamsters (1-7 dpi) and 10 days for recipient hamsters (1-10 days after exposure). Virus loads in the throat swab from the donor and recipient hamsters were measured in VAT cells in a 4-day culture. The viral titers were shown as $TCID_{50}$ from each animal sample. Each open circle represents one animal, and the bar represents each group's median value at the indicated time points.

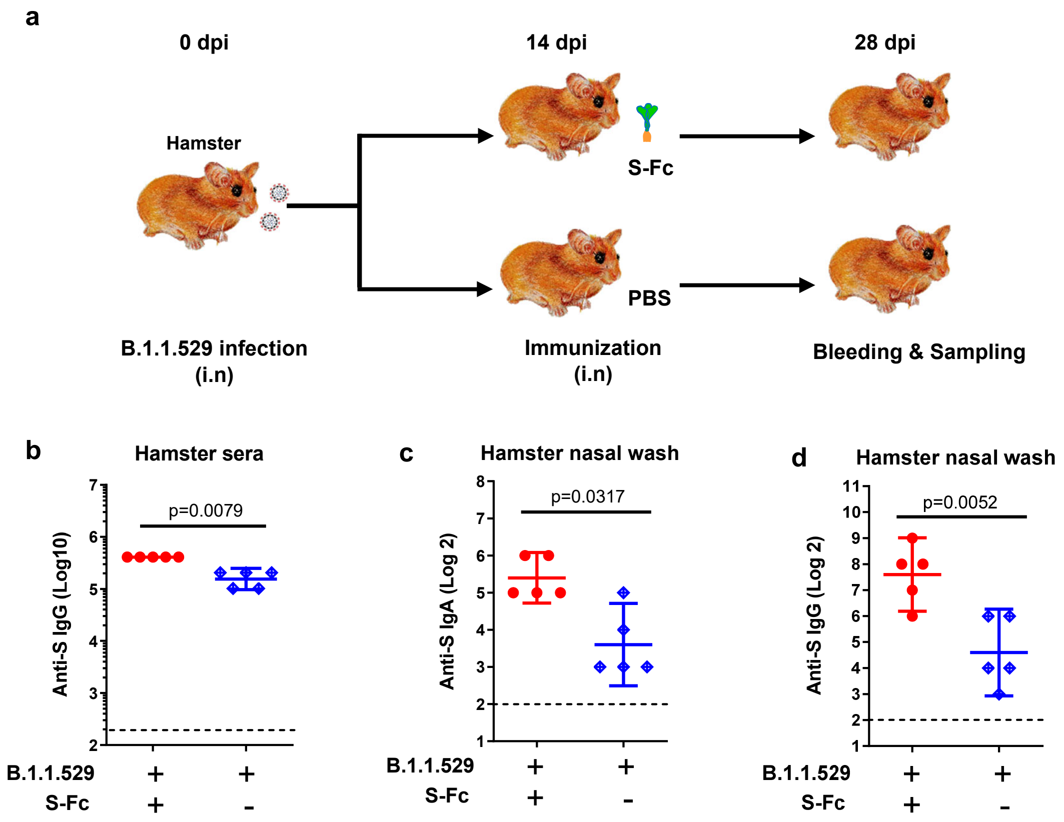

Supplementary Figure 10 The effect S-Fc i.n. immunization on pre-existing immunity. Ten hamsters were i.n. infected with the Omicron B.1.1.529 isolate (1 X10^3 $TCID_{50}$). Two weeks later, five of the infected hamsters were i.n. immunized with 30 μg S-Fc plus 30 μg CpG, while the other five animals received only PBS and CpG (**a**). Two weeks after the i.n. booster, the IgG or IgA levels in the blood, and nasal washes were measured by ELISA and shown as geometric mean with 95% CI (**b-d**). The statistical analyses were performed using the Mann-Whitney test (two-tailed) (**b, c**) or unpaired T-test (two-tailed) (**d**). Dash lines indicate LOD.

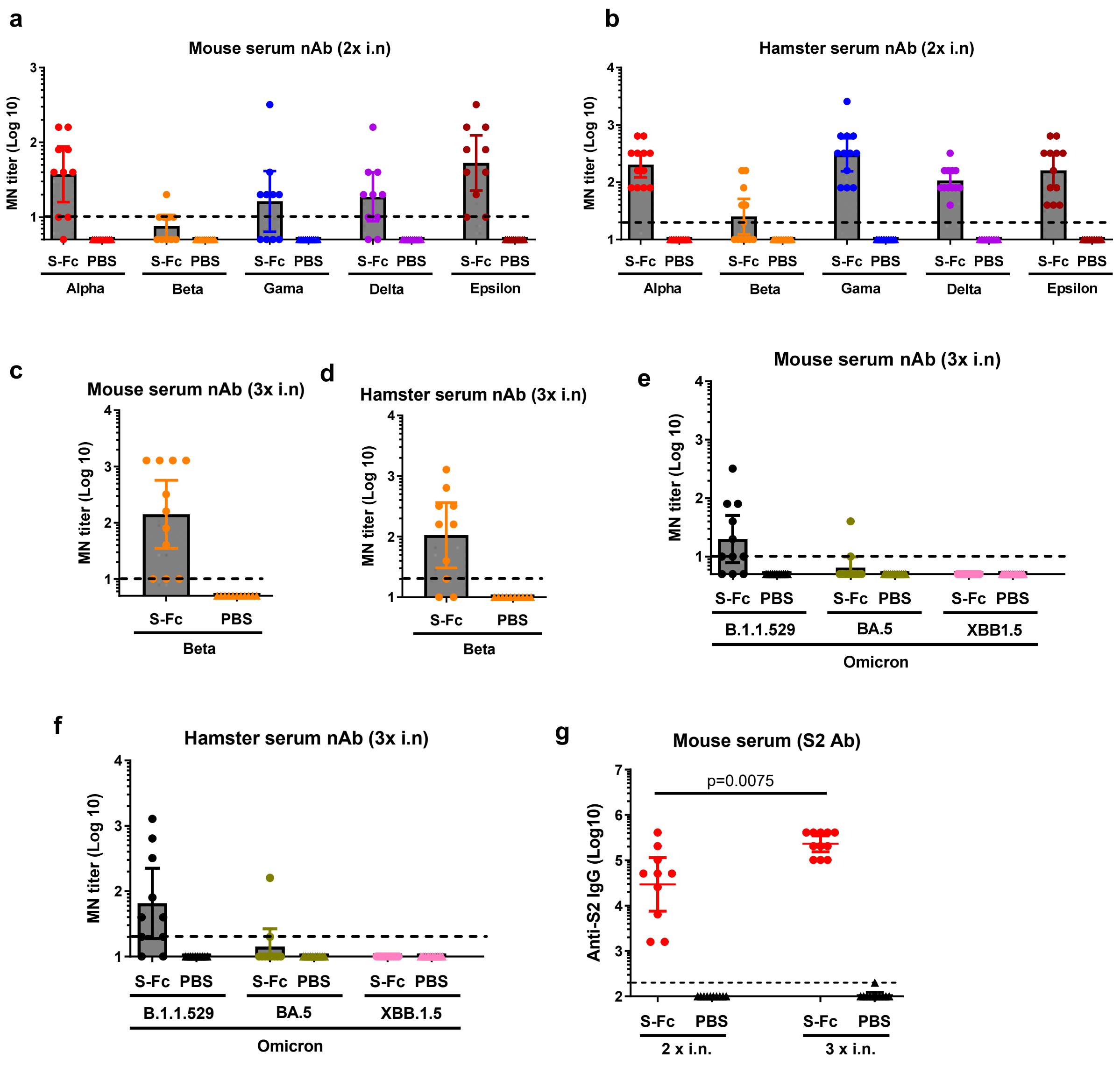

Supplementary Figure 11 a-f Measurement of neutralizing Abs against different SARS-CoV-2 variants or Omicron subvariants. The 6-8-week-old WT C57BL/6 mice (n=10 for **a**, n=11 for **c** and **e**) were i.n. immunized by 10 µg S-Fc, or PBS in combination with 10 µg of CpG); Female hamsters were i.n. immunized with 30 µg of the S-Fc (n=10), or PBS (n=10) in combination with 30 µg of CpG (**b**, **d**, **f**). Both mice and hamsters were i.n. boosted 14 (2x) and 28 (3x) days, respectively, after the primary immunization. Two weeks after the boosts, sera sampled from the immunized animals were heat-inactivated and serially diluted in PBS. The neutralizing Ab activity in the sera was determined by the micro-neutralization test against different SARS-CoV-2 variants or Omicron subvariants. **g Anti-SARS-CoV-2 S2-specific IgG Ab titers in mouse sera**. The S2-specific Ab titers 14 days after the 2nd or 3rd immunization were measured by ELISA using S2 protein to coat the plates. The IgG titers from sera in each

group (n=10 for 2x groups, n=11 for 3x groups) were measured and shown as geometric mean with 95% CI. The statistical differences in the anti-S2 IgG levels between 2x and 3x immunizations were analyzed by an Unpaired T test with Welch's correction (two-tailed). Dashed lines indicate LOD.

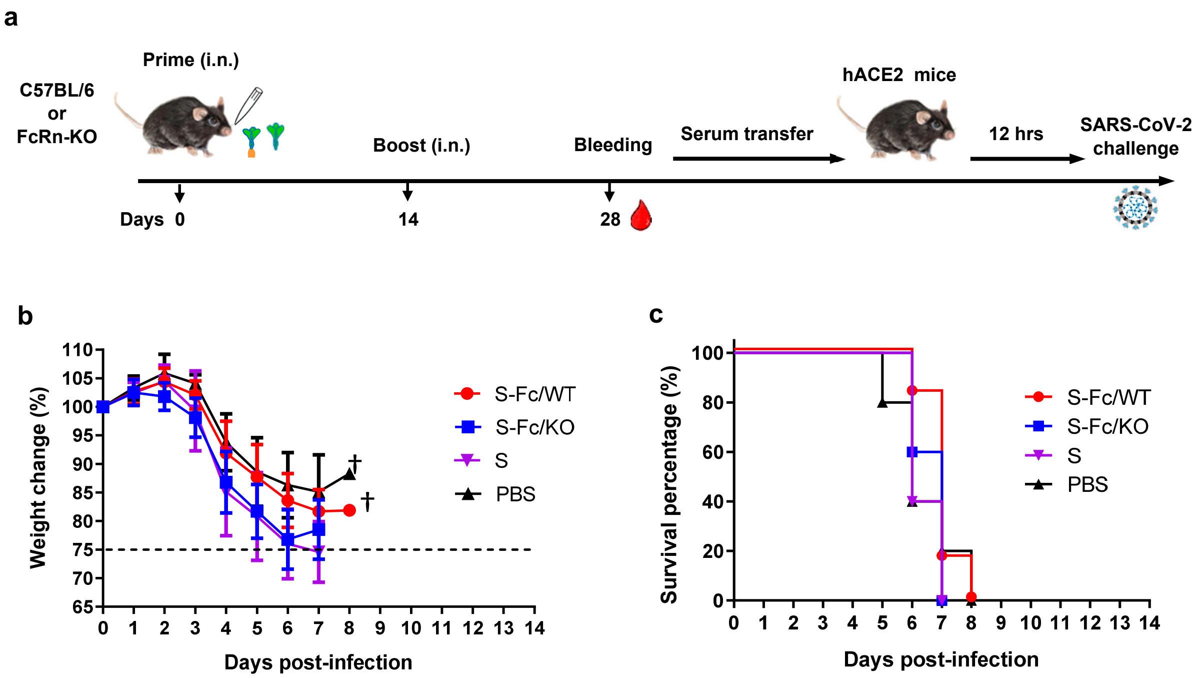

Supplementary Figure 12 Evaluation of the passive protection by serum transfer from the immunized mice. Ten µg of S-Fc, S (with the equivalent molar number), or PBS in combination with 10 µg of CpG was i.n. administered into 6-8-week-old wild-type (WT) or FcRn knockout (KO) mice. Mice were boosted 14 days later after primary immunization. Sera were sampled from all immunized mice, and 200 µl pooled sera from each group were transferred to 8-week-old hACE-2 mice via i.p injection; 12 hours later, all hACE-2 mice (n=5 or 6 /group) were i.n. challenged with ancestral SARS-CoV-2 (2.5 X 10^4 $TCID_{50}$) (**a**) and weighed daily for 14 days (**b**). The survival following the virus challenge is plotted as a Kaplan-Meier curve (**c**). Mice were deceased or humanely euthanized if the humane endpoint was reached.

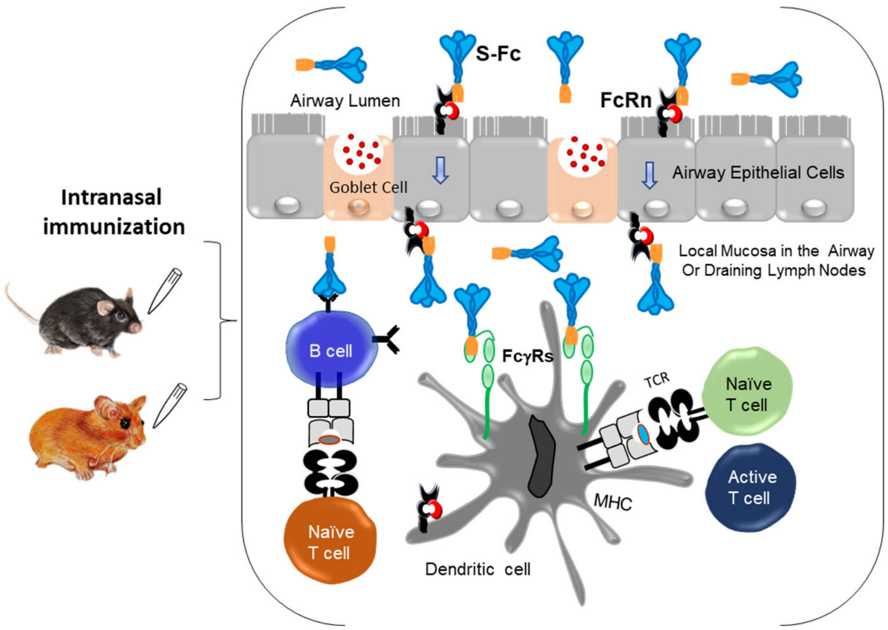

Supplementary Figure 13. Proposed model of FcRn-targeted mucosal delivery of the S-Fc vaccine antigen. The S-Fc proteins are transported by FcRn across the epithelium and targeted to the mucosal antigen-presenting cells (APC), such as dendritic cells. The S-Fc is taken up by pinocytosis or FcγRs-mediated endocytosis by APCs, then processed and presented to T cells in either lamina propria of the respiratory mucosa or germinal centers of draining lymph nodes.

Supplementary Tables

Supplementary Table 1 **a** The infection number of recipient hamsters after exposure to the infected hamsters (donor) was summarized. D: day. **b** Statistical differences in the infection rate among different recipient groups were determined by Fisher's exact test (one-tailed). P: positive number; T: total number. Values marked with asterisks: *, $p < 0.05$.

Recipient	Positive No. after exposure									
	D1	D2	D3	D4	D5	D6	D7	D8	D9	D10
Group 1	2	5	6	6	6	6	5	3	0	0
Group 2	0	1	2	2	2	2	2	1	1	0
Group 3	0	2	2	2	2	2	1	0	0	0
Group 4	0	2	2	4	6	6	6	5	4	4
Group 5	0	5	6	6	6	5	1	0	0	0

Recipient	Summarized case No. (P/T)	P values					
		G1 vs G2	G1 vs G3	G1 vs G4	G1 vs G5	G2 vs G4	G3 vs G5
Group 1	6/6	0.0303*	0.0303*	>0.9999	>0.9999	0.0303*	0.0303*
Group 2	2/6						
Group 3	2/6						
Group 4	6/6						
Group 5	6/6						

Supplementary Notes

1 Intranasal immunization with S-Fc protects old mice from viral infection.

The mortality and fatality of COVID-19 are highly skewed toward older adults, and age is negatively correlated with immune responses after vaccination. The aged mice develop more severe lung damage than the young adult mice upon SARS-CoV-2 infection or re-infection [70]. To assess the immune response and protective efficacy of our vaccine in aged mice, we performed i.n. immunization with two doses of S-Fc (10 μg/mouse) at a 2-week interval (Supplementary Fig. 4a) and used PBS-immunized mice as controls. As shown in Supplementary Fig. 4b, although high SARS-CoV-2-specific IgG was elicited in all S-Fc immunized mice two weeks after the boost, the levels of serum IgG antibodies produced in the aged mice were generally lower than those of the young adult mice. After the immunized mice received a second boost (Fig. 4a), the serum IgG and neutralizing Ab levels elicited in the aged mice were comparable to those of the young adult mice receiving twice i.n. immunizations (Supplementary Fig. 4b, c). We i.n. challenged the immunized aged mice with ancestral SARS-CoV-2 virus (5×10^3 $TCID_{50}$). All aged mice in the PBS control group displayed high susceptibility to virus infection and suffered fast weight loss, resulting in 100% death. In contrast, the S-Fc-immunized aged mice did not exhibit obvious body-weight loss and clinical signs (Supplementary Fig. 4d). All mice were fully protected, leading to 100% survival after the challenge (Supplementary Fig. 4e). Strikingly, no live virus can be measured in the throat swabs of the S-Fc-immunized mice throughout the infection period of 1-6 dpi in comparison to the active virus replication in the throat samples from the PBS control mice. (Supplementary Fig 4f). These results indicated the complete blockage of virus amplification and shedding from the airways of the infected animal by S-Fc immunization after three times immunizations.

2 Intranasal immunization with S-Fc elicits durable protection in mice.

Although the authorized booster vaccinations in adults elicit high levels of neutralizing Abs against the SARS-CoV-2, antibody levels can wane substantially 3-4 months after vaccination [71]. To address whether the i.n. immunization with the S-Fc can sustain long-term protection (Supplementary Fig. 4g), we measured the serum IgG and neutralizing Abs in the hACE2 mouse sera six months after the boost. Most of the immunized mice maintained a significant level of S-specific IgG Ab (Supplementary Fig. 4h) and the neutralizing Ab activity (Supplementary Fig. 4i) in their sera compared to PBS control animals, although the levels were lower than those in the immunized sera collected two weeks following the boost. To investigate long-term protection, we further challenged mice with ancestral SARS-CoV-2 virus (2.5×10^4 $TCID_{50}$) six months after the boost (Supplementary Fig. 4j). Following the challenge, mice immunized with the S-Fc exhibited significantly reduced viral replications in nasal turbinate, lung, and brain tissues (Supplementary Fig. 4j), while the PBS control mice displayed a considerably higher level of viral replications. Overall, intranasal delivery of the SARS-CoV-2 vaccine engendered an effective and long-term memory immune response and provided sustained protection against challenges.

3 Intranasal immunization with S-Fc protects hamsters from viral infection

Golden Syrian hamsters are highly susceptible to the SARS-CoV-2 virus and exhibit disease symptoms like humans, including severe lung inflammation [68]. We showed that hamster FcRn bound human IgG1 and the S-Fc (Fig. 1C). Hence, hamsters were i.n. immunized with 30 µg of the S-Fc protein or PBS together with 30 µg CpG and boosted 2 weeks later (Supplementary Fig. 7a). As shown in Supplementary Fig. 7, the S-Fc immunized hamsters induced significantly higher levels of IgG (Supplementary Fig. 7b, $p<0.001$) and neutralizing Ab (Supplementary Fig. 7c, $p<0.001$) titers in comparison with the PBS-immunized hamsters. Next, we investigated whether hamsters i.n. immunized by the S-Fc resist to SARS-CoV-2 infection. Groups of hamsters (8 hamsters/group) administered intranasally with S-Fc or PBS were challenged with 1x

10^6 $TCID_{50}$ of ancestral SARS-CoV-2 virus 17 days after the boost. Hamsters within the PBS group underwent mild to moderate body weight loss (Supplementary Fig. 7d), but none succumbed to viral infection. In contrast, the S-Fc-immunized hamsters did not show weight loss (Supplementary Fig. 7d). Meanwhile, the S-Fc immunized hamsters had significantly reduced the number of viruses in nasal washes compared to that of PBS-immunized mice at 2 and 4 dpi (Supplementary Fig. 7e*, $p<0.01$-0.0001*), suggesting the decline of virus shedding from these animals. Third, we measured viral replications in the nasal turbinate, trachea, lung, brain, and intestine 5 dpi (Supplementary Fig. 7f). High titers of live SARS-CoV-2 virus were detected in the nasal turbinate and lung tissues of the PBS-immunized control hamsters; on the contrary, no live virus was isolated from the nasal turbinate and lung tissues of animals who received i.n. immunization with the S-Fc. Hence, virus titers in the nasal turbinates and lungs of the animals that received the i.n. immunization on day 5 post-infection were significantly lower than the virus titers in the animals that received PBS at the corresponding time postinfection (Supplementary Fig. 7f, $p < 0.05$) for the virus titers in the nasal turbinates and lungs, respectively. In addition, we did not find a live virus in the trachea and brain tissues of either PBS- or the S-Fc-immunized hamsters. These data indicate that the i.n. immunization with S-Fc provides protective immunity against SARS-CoV-2 infection in hamsters.

To examine the efficacy of S-Fc for boosting pre-existing immunity conferred by infection with a heterologous SARS-CoV-2 variant, we intranasally infected 10 hamsters with the Omicron B.1.529 strain (1 X10^3 $TCID_{50}$). After two weeks, we i.n. immunized five infected hamsters with S-Fc plus CpG, while the other five received only CpG. Two weeks after the boost, we measured their IgG or IgA levels in the blood samples and nasal washes from all the animals. As shown in Supplementary Fig. 10, the S-Fc intranasal immunization significantly boosted pre-existing serum IgG levels and the levels of nasal IgG and IgA from the hamsters previously infected with the Omicron B.1.529 strain.

4 Intranasal immunization with S-Fc induces neutralizing Abs in mice and hamsters

SARS-CoV-2 constantly evolves, resulting in variants of concern (VOCs). Omicron subvariants are not only highly contagious but also resistant to nAbs. To show whether the sera from the mice or hamsters that were i.n. immunized by S-Fc neutralize SARS-CoV-2 variants, we further conducted a neutralization test. The results showed that mice or hamsters that received two doses of intranasal immunizations with S-Fc in a two-week interval were able to neutralize SARS-CoV-2 strains, α, β, γ, δ, and ε, as compared to the control group (Supplementary Fig. 11a, b). Interestingly, the level of nAbs against strain β was lower in the immunized animals. However, after receiving three doses of i.n. immunizations, most mice and hamsters developed higher levels of nAbs against strain β (Supplementary Fig. 11c, d).

We further examined whether animal sera of 3-dose intranasal immunizations could neutralize Omicron variants. Although most immunized mice or hamsters developed nAbs against B.1.1.529, they did not produce strong Abs against BA.5 or XBB.1.5 (Supplementary Fig. 11e, f). These results indicate that Abs induced by ancestral SARS-CoV-2 spike antigens are ineffective at neutralizing Omicron BA.5 or XBB.1.5.
